# Passivity, being-with and being-there: care during birth

**DOI:** 10.1007/s11019-016-9686-5

**Published:** 2016-02-22

**Authors:** Tanja Staehler

**Affiliations:** University of Sussex, Falmer, Brighton, BN1 9QN UK

**Keywords:** Care, Childbirth, Homebirth, Passivity, Waiting, Heidegger, Discourse, Communication

## Abstract

This paper examines how to best be with women during birth, based on a phenomenological description of the birth experience. The first part of the paper establishes birth as an uncanny experience, that is, an experience that is not only entirely unfamiliar, but even unimaginable. The way in which birth happens under unknowable circumstances (in terms of when, how, with whom…) creates a set of anxieties on top of the fundamental anxiety that emerges from the existential paradox by which it does not seem possible for a body to give birth to another body. Would homebirth provide a remedy to the uncanniness? The result yielded by medical studies is confirmed by the phenomenological perspective taken here: homebirth might be reassuring for some, but not for everybody; choice of birth place is important. Once the birth process starts happening, another layer of strangeness is added: it turns out to be an experience of radical passivity and waiting, normally. The question thus becomes how to best care for somebody who is exposed to uncanniness, passivity, and waiting. Martin Heidegger’s concepts of care and discourse prove useful in examining how to facilitate rather than interrupt this process. It becomes necessary to think beyond verbal communication towards a wider concept of communication that involves silence and intercorporeality. Birth requires a special kind of being-with as being-there.

The birth process is undoubtedly a very unusual experience. This experience will be approached here from the phenomenological perspective, meaning that a description of the experience or of what it is like to give birth will be attempted. The purpose of this description is to explore how to best be with a woman who is giving birth.

The phenomenological description yields strangeness or unfamiliarity as the pervasive character of the experience. While this result is not as such surprising, it will be helpful to examine this strangeness more closely with respect to its different dimensions. Such differentiation will make it possible to see how even the more mundane dimensions of the experience become affected by the fundamental uncanniness of birth as an experience that is unimaginable. Furthermore, the uncanniness of birth is exacerbated by the fact that passivity and waiting are normally a crucial part of the process. The question thus becomes how to best be there for somebody who is exposed to passivity and uncanniness.

In caring for a woman (or couple) undergoing this uncanny experience, it is significant to consider the radical clash between the perspective of the woman facing this experience for the first time (or, if not first, in any case as something that always remains unknown) whereas the midwife undergoes this experience from the outside every day. Taking the other’s perspective is thus crucial. But at the same time, if the care works out well, caring for somebody in such a limit situation can make the carer appear like an angel: a super-human guide and guardian. In the best case scenario, midwives reassure us that miracles (or what seems like one due to its inconceivable character) are possible. In the worst case scenario, they can make us wanting to escape: from the situation and from our bodies, neither of which is possible.

There are several factors contributing to a good birth experience, and our focus here lies especially with the situational and communicative ones. If the character of the experience is determined by uncanniness, one possible response appears to be homebirth, or making oneself at home to counter-act the strangeness of the experience. In the case of homebirth, midwives are crucial. Yet it turns out that if the relationship between midwife and mother or couple works well in terms of communicating within and responding to the situation, the question of place becomes secondary: a caring midwife can make us feel at home in such a way as to override the surrounding circumstances. While choice of birthplace is important, the quality of care is more important for alleviating fears than the question of home versus hospital.

## An uncanny encounter

Any pregnant woman will to some extent imagine the birth process. That is a normal attempt at coping with an unfamiliar experience, and it is a useful attempt since it helps making some crucial decisions, including the decision as to where this experience should take place. Our first step will be a closer exploration of the dimensions of strangeness that characterises birth.

- *When* the birth happens is unknown. From a certain point of pregnancy onwards, it could be basically any day, any moment. There is thus a constant sense of uncertainty which makes itself present especially around any deviations from the everyday, such as travels.^1^
- *Where* the birth happens is unpredictable, and yet needs to be pre-selected. In other words, it is necessary to decide between the locally available possibilities (hospitals, birth centres or birth houses, homebirth) while still bearing in mind that in the end, it might nonetheless be an entirely different place. A birth that starts at home might be moved to the hospital, a planned hospital birth might end up happening at home, and that is not to mention more unusual locations like vehicles of transportation.
- *Who* will be present is uncertain, and yet very much worth pondering. For example, if the father is supposed to be present, it is worth imagining and discussing whether both sides are comfortable with this, and what his role would be, bearing in mind again that there are limits to anticipating the actual course of events. A major factor of uncertainty concerns the health professionals present: normally, midwives. Under most circumstances, the midwives encountered in the situation will be strangers, and it is worth mentally preparing for this.^2^
- The *gender* of those present is unknown, and especially of healthcare professionals: a male midwife would initially be a shock to most birth-giving women.
- *How* the birth happens is unforeseeable. Will the process be long or short? Will it require medical interventions or not? There are lots of unknowns, and some of them deserve imaginative engagement. For example, does a water birth seem desirable or not? If the process is prolonged, where and how should the waiting happen? What would my ideal birth look like, and what is completely unfathomable for me? How can these findings be conveyed in a birth plan? Despite the need and usefulness of planning, there are many dimensions that remain unforeseeable since I cannot anticipate what my body will feel like during the process, and to what extent this undermines my previous intentions and plans.
- *How* the birth will be *possible* is unimaginable. In addition to the factors of ‘how’ mentioned under (e), there is a deeper dimension to the unforeseeableness of how the birth will happen. This is not a matter of how exactly will it happen, as in the previous point, but more a ‘how on earth will it happen’? The most fundamental problem is that birth as an event is unimaginable. I cannot imagine my body giving birth, I cannot imagine that such an event is possible. And even after it has happened to a person once or more, there is still some level on which the experience remains inconceivable.

Birth is a paradox of sorts because it is in some sense impossible (namely, unimaginable) and yet at the same time, entirely possible (because we know it happens all the time). At this point, the unimaginable character of birth will be explored further with the help of Martin Heidegger’s concept of the uncanny that elucidates how humans tend to deal with uncanniness— namely, by creating higher levels thereof. Heidegger explains this tendency through a line from Sophocles’s ancient tragedy *Antigone*. In the first choral ode of *Antigone*, the nature of the human is designated as the most uncanny (Greek *deinon*) among many uncanny things. Our relation to uncanniness is relevant because it is a dynamic one. We experience the world as uncanny on some basic level because we did not bring it about, but were born or ‘thrown’ into it, and into a particular time and space, without ever being able to fully grasp this uncanny world. However, we do not stop at this encounter with the world as uncanny, but try to make ourselves at home in the midst of this uncanniness. As we do so, we create higher levels of uncanniness. We strive to make ourselves at home in the midst of that which overwhelms us, and yet, we ourselves have a tendency to overstep the boundaries of the familiar. In this respect, human nature is excessive. Humans surpass “the limits of the homely” (Heidegger 2000, p. 161). According to Heidegger, this excessive nature is best characterized by the Greek term *techne*, translated as craft, power, or violence.

One of the most important overwhelming powers against which humans react by creating higher levels of uncanniness is nature. In the choral ode, nature is represented by natural elements like the earth and the sea, which have inexhaustible energies and confront us with their waves, circles, and cycles. Exposed in this fashion, we do not give up; we rise to the challenge, making ourselves at home in the midst of that which overpowers us. One crucial manifestation of our link to nature that confronts us with uncanniness is illness. Illness will be used here as a general example for uncanniness; this is not to imply in any way that pregnancy is an illness (yet childbirth as well as illness involve healthcare professionals). Illness means that our bodies fail us, as it were, and become affected in detrimental ways by internal or external factors. Illness is particularly uncanny because the affected individual normally does not understand it (at least not fully), yet depending on the extent of the illness, it can affect our existence wholly and to its core. The craft or *techne* that humans engage to fight illness is medicine which was known already to the ancients, but began taking on its natural scientific shape in the seventeenth century. Medicine thrived in the twentieth century, and it was at that time that births started taking place in hospitals.

When the possibility of giving birth in a hospital became historically a widespread possibility, this certainly meant a significant reduction of fatalities for birth-giving women as well as for infants. Yet at the same time, it created a new level of uncanniness, in line with Heidegger’s description of the human tendency to evoke higher levels of uncanniness in combating previous uncanny phenomena. Medicine in general fits the Heideggerian description as a higher level of uncanniness that replicates some of the features which make illness so uncanny. Just as we do not usually understand illness, we also tend to not comprehend medical treatments. Medical professionals are not always eager to explain, and the facts are usually quite complicated indeed. Yet at the same time, medicine affects our existence fundamentally. When I ‘take’ my body to the doctor and experience discomfort at the realization that she might indeed only be interested in my body as a physical object, this unease creates an opportunity for the phenomenological discovery that I *am* my body rather than merely having a body, and that my body is lived and experienced rather than a merely physical body.

It will not be possible to examine here the character of medicine in general and possibilities of remedying the discomfort often felt in the face of its uncanniness. Yet the topic has some direct implications for birth. The pain involved in the process and the possibility of complications create an affinity between childbirth and illness, but at the same time, it is often and rightfully pointed out that birth is not as such a medical condition and should not be treated in this fashion. Particularly in terms of uncanniness, the hospital atmosphere can be fear-inducing rather than fear-alleviating.^3^ There has been a general tendency to create a ‘homey’ atmosphere in maternity wards and remove or conceal equipment of medical technology as much as possible. Nonetheless, a hospital is a hospital, and the desire to return to homebirths is indicative of this awareness.

How do homebirths fare when it comes to uncanniness? An important 2011 study of the UK situation conducted by Peter Brocklehurst showed that homebirths do not increase the likelihood of birth complications. However, the case of women giving birth for the first time (“nulliparous”) differs from that of women who have given birth at least once before (“multiparous”). For nulliparous women, the danger of stillbirths and perinatal injuries were slightly, but indeed only minimally,^4^ increased for homebirths; for multiparous women, they were not. Yet the most substantial difference between nulliparous and multiparous women concerned transfers to hospital which were necessary for about 40 % of nulliparous women (vs. ~10 % of multiparous women). For both groups, the likelihood of medical interventions was significantly lower in the case of homebirths.

Brocklehurst thus concludes that his “results support a policy of offering healthy women with low risk pregnancies a choice of birthsetting” including homebirths and non-obstetric midwifery units (Brocklehurst 2011, p. 1). Brocklehurst’s recommendation coincides with the phenomenological perspective because being able to choose the most desirable situational context for the uncanny experience of birth can be very helpful. On the basis of the phenomenological exploration undertaken here, it appears that imagining the birth situation is a useful exercise, despite the aforementioned limitations of such thought experiments. One of the most important decisions to be made concerns the location or ‘where’ of birth, and being given a choice already contributes to a sense of participation rather than determination from the outside.

Perhaps the phenomenological perspective developed here points in the direction of a homebirth? Yes and no. Initially, it might seem that one’s home was the preferable place for an uncanny experience since an unfamiliar space increases the sense of strangeness. Furthermore, a hospital with medical technology might evoke an uncanny atmosphere from the outset. Yet already the fact that more than a third of all nulliparous women who planned a homebirth need to be transferred to the hospital shows that the situation is more complex. Brocklehurst’s study is purely quantitative. This has the advantage of high reliability for the English context since data from all participating NHS units between April 1st 2008 and April 30th 2010 was considered. Yet at the same time, due to the quantitative nature and complete lack of interviews, the study provides no explanations or interpretations. Indirect results from qualitative studies on birth in general^5^ along with common sense yield three general categories of reasons for these transfers: medical reasons (i.e., the need for medical interventions, including caesarians and forceps deliver); pain relief (epidural); psychological reasons. Psychological reasons include fear and anxiety, and they can contribute to a need for medical or pain relief interventions. In turn, being in a home environment which makes medical interventions and epidurals impossible can certainly enhance fear and anxiety. As one of my colleagues once put it: “Call me a wuss, but I like to be around life-saving equipment when I give birth!”

At the same time, the data provided by Brocklehurst with the almost null difference in detrimental results shows that the decision for a homebirth creates very little actual risk, and for multiparous women, actually no risk at all, but a smaller likelihood of interventions. In that sense, the difference might well be a psychological or ‘felt’ one. Midwives who arrive for a homebirth carry life-saving equipment, and in that sense, even somebody who prefers to be around life-saving equipment when giving birth does not need to exclude homebirths. Yet from a phenomenological perspective, the ‘felt’ difference which is undoubtedly a factor in transfers from home to hospital must not be neglected. Among the likely reasons would be the realization that there is more pain involved than anticipated, but also that the birth-giving body feels different and more alien than presumed. It should also be acknowledged that midwives coming to the house normally work in shifts, and when a shift is over, there are new midwives taking over just as it would be the case in a hospital. Furthermore, in addition to the possibility to be transferred into hospital for the birth, which we have seen to be a frequent occurrence for first time mothers, there is the risk of needing to be transferred after the birth, for repairs (e.g., episiotomy), which is more disruptive to the mother-infant-bonding experience than being in a hospital to begin with. It should also be noted that homebirth is only safe for the infant in countries where transferring the infant to a hospital for any complications or concerns is easy and fast.

Yet there are other factors as well, and some of them might well be open to change while others can be anticipated beforehand by an exercise in the imagination. One aspect of birth that is crucial and yet often neglected is the significance of waiting. It is a common yet mistaken assumption that the birth process simply gets more and more difficult as it advances. Rather, the final phase is often easier than the previous one, already because the end of the process is coming tangibly closer and because being able to participate (by ‘pushing’) is in certain ways easier than passively waiting. The significant role of waiting makes it advantageous to prepare for some entertainment or other accessories. That is where the home context seems to provide many advantages; yet an often disregarded factor needs to also be taken into account: midwives. In general, if the preference for the home is motivated by the desire to maintain a familiar context and atmosphere in preparation for an uncanny encounter or experience, it must be noted that the arrival of midwives into the home context will in any case mean an encounter with strangers. Only under exceptional circumstances will the midwife be a familiar one^6^; in imagining the situation, it should thus be imagined as an encounter with a stranger.

Admitting a stranger into my home creates a situation that calls for hospitality, as we learn from Jacques Derrida,^7^ but also from common sense. Such hospitality is certainly noticeable and concerns mundane dimensions such as sustenance,^8^ but also the choice of entertainment.^9^ Furthermore, if the ‘guests’ are behaving in a disruptive fashion, their presence in the home becomes more noticeable than in a neutral setting. It might seem strange that such mundane hospitality would have any impact on the birth experience. Yet if we consider the tension between mundane and much deeper existential dimensions that determines the birth situation in general (on the affective level as well as otherwise), this influence might become more understandable. The confrontation with an uncanny experience in a mundane setting can make certain aspects of that setting entirely irrelevant, but other aspects might suddenly become more problematic or pressing. Being sociable, communicative, or hospitable is difficult under the circumstances.

Moreover, the tension between mundane and fundamental levels plays out for hospitality as well. There is a level of fundamental hospitality involved in the birth experience.^10^ This fundamental hospitality designates the need for me to let the midwife take care of my body as my most basic and irreplaceable home. It requires me to trust her and let her enter this home of mine, in the general, but also in the literal sense. If there are problems on the level of mundane hospitality, it also becomes more difficult to achieve this fundamental hospitality. In other words, even though concerns on the level of mundane hospitality might appear trivial, they can be disruptive in relation to the more fundamental level of giving the midwife access to my body, especially since such a major existential situation is at stake here.

When it comes to an imaginative engagement with homebirths, hospitality thus needs to be part of the imaginative scenario as well as the potential need to be transferred. However, if the decision is nonetheless for a homebirth, the possibility of transfer should in the situation itself better not be too much on the woman’s mind. Considering other options creates a general atmosphere of uncertainty or indecisiveness that is unhelpful. One of the biggest advantages of homebirth is the possibility to stay put, in all senses. Once the baby is born, everybody is immediately there, home, where they can stay, recover, and celebrate together—and the home is home again, just even more so, substantially enriched by the new arrival.

Overall, we have seen that there can be advantages as well as disadvantages to a homebirth, and the need for an imaginative engagement with birth thus gets confirmed. Such exercises in imagination should also include a dimension of birth that is crucial yet often neglected, partly due to the temptation to focus immediately on the crucial point of the process: the actual birth, that is, the appearance of the baby. But a substantial part of the process is determined by passivity and waiting, and those are difficult to deal with in their own right, but even more so when waiting for something fundamentally uncanny to happen.

## Passivity and waiting

Passivity is something we neither value nor consider much. A phenomenological description of birth shows that this is a mistake. However, when we initially explore birth, the same inhibitions emerge that determine our relation to passivity in general. Just like it is a negative characteristic to describe somebody as “passive”, there is also an impression that a more active engagement is conducive to giving birth. The “Active Birthing” movement symbolises this conviction. Passivity in birth is usually associated with hospital births and more precisely, medicalised birth. By contrast, qualitative research on birth as inspired by existential phenomenology tends to focus on the woman as “subject” rather than treated “object,” where “subject” is often interpreted in terms of autonomy and activity.^11^

The existential phenomenology of birth presented in this article places an emphasis on passivity and thus counters the everyday as well as current research emphasis on activity in birth. This is not to deny but merely to supplement the significance of active birth. A few preparatory clarifications are in place. Firstly, a general reminder concerning the position taken here: it is not our purpose to argue against hospital births, as should be clear from the previous section. The result of our considerations was merely to encourage imagining and give women freedom of choice concerning their preferred place of childbirth.^12^ Secondly, our purpose in highlighting the significance of passivity is not to somehow indicate that “doing nothing” would be the best attitude during birth. Rather, passivity is an essential component of the experience, and for this reason, it is helpful to consider how to deal with passivity. Importantly, neither is passivity nothing, nor does it mean doing nothing. Passivity is complex and multi-faceted, and responding to an experience of passivity is quite difficult, much more so than doing nothing. While passivity describes a general attitude of ‘letting-be’ and applies to childbirth as a whole, there is a more concrete part of childbirth that requires passivity, but can also be prepared for more concretely: waiting.

Waiting is crucially a temporal phenomenon, and our body plays no intrinsic role in it.^13^ A general phenomenology of waiting (as can obviously not be accomplished here) would begin with the difference between objective time and phenomenological time, or clock-time and time as experienced. Waiting generally designates that time stretches, or that it passes painfully slowly. Ten minutes of waiting always feel longer than ten minutes of a normal activity. Even waiting for something exciting does not change the fact that the flow of time is conceived as too slow, expressed by the formulation “I can’t wait (for X to happen)”. On the other hand, if the event waited for causes fear, there is nonetheless waiting as being obsessed with the flow of time or its seemingly standing still. Birth will normally involve both of those dimensions of waiting: waiting with excitement for the baby, but also waiting with fear for the further development of the process.

In waiting, time expands, and nothing in the world provides a remedy. Nothing? In normal experiences of waiting, we often wish we had brought a book, or a conversation suddenly provides distraction. During labour, conversations are difficult and not very desirable. But other forms of distraction can well be helpful especially during the initial stages. It might indeed not be a bad idea to bring a book.^14^ The more widespread response to a situation of waiting is television. Many women indeed report having spent some of the initial part of the birth process watching TV (at home or in the hospital), and those who do not want to be subject to the unpredictability of regular TV might want to include the question of visual entertainment in their preparatory imaginative engagement by sorting out a DVD, etc. Of course, this depends on whether and what woman likes to watch when discomforted.

Admittedly, the moment will come when distraction through entertainment is no longer possible, and that is when the body makes itself so present that a kind of Cartesian mind–body-dualism becomes desirable. In other words, there is a strong desire for body and mind to be detachable in such a way that the body could be left, at least momentarily, by that part of us which is responsible for thinking, feeling, and experiencing. Of course, there is no experiencing without body, and no thinking and feeling either, though the latter might be less obvious. Pain thus undermines Cartesianism on the level of experience rather than on the logical level, and it undermines it exactly because dualism would be desirable in such a moment yet remains all the more so impossible.

But how to best assist somebody who is facing an inevitable yet uncanny experience, and one that involves waiting and passivity? If the time of waiting has not robbed the woman of all energy and determination, the final phase becomes manageable exactly because it is clear that it is the final phase. Therefore, creating conditions for ‘good waiting’ and ‘good passivity’ is crucial. Others are crucial in this respect because others affect us most. Others have always already affected me and will continue to affect me. Since birth is a particularly volatile situation, it is crucial to understand better how others affect me, especially on the level of care and communication.

## Care and discourse

How can others best help us dealing with the affects in this extra-ordinary situation? The current article proposes that Heidegger’s philosophy is most helpful in this respect. Both care and discourse are important dimensions when it comes to the experience of birth. Midwifery is a care profession. Starting from etymology (Latin *cura*) and common sense understandings, Heidegger makes it clear that the everyday notions of ‘concern’ or ‘worry’ will not lead us to the primordial meaning of care. The deepest meaning of care is ontologically so fundamental that it becomes synonymous with existence. Our existence is care because our existence is an issue for us (Heidegger 1978, p. 42); we do not just live our lives, but reflect on it.

While his overall sense of care refers to the world in general and also our dealings with objects, they can be shown as relevant for healthcare relations.^15^ For our purposes, his considerations regarding *Fürsorge*, ‘care-for’ (Heidegger 1978, p. 122), are particularly important. Heidegger explains that welfare and care professions become necessary exactly because we for the most part do not sufficiently care for each other, but live alongside each other, treating each other like, or almost like, objects in the world. In contrast to this indifference, there are two ‘positive modes’ of care, though ‘positive’ initially just means that caring-for is happening, not that it is necessarily happening in a positive or helpful fashion. The first, much more common form of caring-for consists in ‘leaping in’ for the Other (*einspringende Fürsorge*), taking the care away from him or her (Heidegger 1978, p. 122). While this may sound helpful especially on a misplaced understanding of care as worry, it proves detrimental because it turns the Other into somebody “who is dependent and dominated even if this domination is a tacit one and remains hidden from him/her” (ibid.). The second form of caring-for, in contrast, means to ‘leap ahead’ of the Other (*vorspringende Fürsorge*), not taking his or her place, but giving the care back to the Other. This caring-for treats the Other not as a “*what* which it takes care of” but strives to help the Other “to become transparent to himself [or herself] in his [or her] care and free for it” (ibid.).

This distinction has direct implications for midwives and health professionals helping in the birth process. Although Heidegger certainly does not discuss this example, it would be true to say that birth (just like anxiety) individualises: nobody can give birth for me. Nonetheless, the way in which the situation of birth creates a dependence on others makes it tempting to wish that the Other could ‘leap in’ for me. Yet this leaping in cannot ultimately be helpful. Once it feels like the process is out of my hands and I no longer conceive of myself as the most crucial participant, it becomes tempting to treat myself or think of myself as a helpless object. Conceiving of myself as an object is relatively easy, due to my split nature of being subject/object which Heidegger, to avoid misunderstandings easily created by this traditional terminology, re-conceives as our being ‘thrown project’: I am thrown into the world like an object, yet I am also a project of multiple possibilities. Care means having to negotiate myself as thrown project.

Abnormal states like sickness or pain easily create a situation in which my self-perception changes in such a way that I would like to distance myself from my body. Pain is existentially threatening exactly because it makes me aware that I am unable to escape. Being (rather than having) a body means that existence is a ‘no way out’ situation, as far as my corporeality is concerned. As a result, ‘leaping in’ care is particularly dangerous in the health profession: the patient all too willingly accepts the invitation to take further distance from the already alienated body and expect for the Other to take over. While such trust in professionals appears helpful in cases of sickness where actual medical intervention is necessary, it can only be helpful in childbirth if the situation indeed takes a turn to the problematic and thus medical. For ‘normal’ birth, ‘leaping in’ creates problems since it will become obvious quite soon that nobody can give birth for me; so I have to accept this body back. Much more helpful and less frustrating is thus a form of care that ‘leaps ahead’, trying to free myself for taking care of myself and helping me to realise that the responsibility is indeed mine.

How can this best be achieved? Heidegger does not provide precise guidelines, but it becomes obvious that it is a matter of relational attitude. In order to examine my relation to the other more closely and see how different attitudes manifest themselves, it will be useful to invoke the second major dimension of existence in which being-with others happens: discourse (*Rede*).

Discourse is very significant in the healthcare profession. Before attending to some crucial moments of Heidegger’s analysis, I would like to give a few examples from women who felt they had been failed by health professionals in childbirth. One woman reported: “I was more or less an object, not a human being but something they had to get something out of, nobody told me anything, nobody said a word, no explanations nor any kind of information whatsoever” (Lundgren 2011, p. 124). The communication problems experienced by this woman are obvious; yet a remedy to the situation is much less straightforward than it may first seem. Simply speaking to women is not sufficient, though it sounds as if the lack of speech was the biggest issue in the account just given. But a different woman reports: “they just told me how I was feeling” (Lundgren 2011, p. 141). This example shows how speech can itself be quite patronising, like an extreme form of leaping in.

A brief example from my own experiences indicates further how complex the situation is. The midwives who had come to attend my homebirth kept asking me every few minutes ‘How are you doing?’ A perfectly friendly, everyday question, and indeed a question, not a patronising form of discourse. Nonetheless, this intervention was experienced by me as unhelpful after a short period of time because the question was repeated over and over again, every few minutes. Initially, I responded ‘I’m okay, given the circumstances’. Later, I just said ‘I’m okay’, then just ‘okay’. I realised that I did not want to be forced into the land of words or verbal language every few minutes, especially since there was not much to say and little need to speak. The incessant questions turned me back into a thinking and speaking subject when I wanted to settle into the situation as a body, trying to feel out how to best be a body under the circumstances: waiting, passive.

What is called for is thus a wider notion of discourse as a mode of being with the other without relying solely on words. Such a wider notion then also makes it possible to integrate words and use them in a reassuring, supportive rather than disruptive or even patronising fashion. Heidegger provides such a concept of discourse in section 34 of *Being and Time*. Three characteristics in particular are worth noting, for our purposes. Firstly, Heidegger points out that discourse is the foundation of language (Heidegger 1978, p. 161). Discourse, and thus the relation to others whom we address (or refuse to address) is the more primordial phenomenon in comparison to verbal language. Our usual restricted understanding of language as concerned with conveying information comes about because we do not acknowledge that verbal language is derivative from discourse in the wider sense. This wider sense includes gestures, mimics, sounds, etc. Secondly, Heidegger insists that we never just hear sounds, but hear specific entities, even though we might be mistaken about what exactly we hear. We immediately give meaning to what we hear; we make sense and are affected by this sense. Especially in situations of vulnerability, this relation needs to be considered. Thirdly, “hearing and keeping silent are possibilities belonging to discoursing speech” (Heidegger 1978, p. 161). Silence is not a lack of speech, but a genuine and very important dimension of discourse.

Listening to the Other and listening to the situation means not being afraid of silence. If such discourse in the wider sense is successful, it “brings about the ‘sharing’ of being attuned together and of the understanding of being-with” (Heidegger 1978, p. 162). As Heidegger also points out, I cannot take the Other’s fear away by fearing for them, but there can nonetheless be an understanding of how the other is affected, and in that sense, a ‘sharing’ of affects. This does not mean to take away the Other’s fears and anxiety, but help them to not get overwhelmed by them. “Listening to each other, in which being-with is developed, has the possible ways of following, going along with, and the privative modes of not hearing, opposition, defying, turning away” (Heidegger 1978, p. 153).

What does this mean for birth-related discourse? Discourse is certainly not limited to the words that are being spoken. Leaping-ahead care, for midwives, can consist in breathing with the woman, nodding in support, providing a massage if desired, giving support to the body: being-with that goes beyond words. It is crucial for the midwife to *be there: da sein*. Such a wider concept of discourse could also be referred to as intercorporeality. Discourse which involves silence, hearing, and letting-be allows the woman to stay a ‘who’, that is, own up to being and remaining a ‘who’ in the situation, even if she is not a discourse partner in the ordinary sense.^16^ It means supporting passivity rather than interrupting it, acknowledging the existential precariousness which makes even the most ordinary mundane problem suddenly complex, and listening to what the woman needs, no matter how difficult it may be for her to communicate it. Of course, the emphasis placed here on silence is not meant as a categorical demand to be without words; midwives can also speak to reassure as well as to inform, and that is important. But equally significant are the willingness to be silent and the understanding that a response from the woman in labour should not be expected.

## Conclusion

On the basis of the reflections on the experience of birth and the challenges it poses for care and discourse, we can conclude that the quality of care matters more than the birth setting (home or hospital). While it is very useful to have homebirth available as an option for those women whose fear of the unfamiliar is alleviated by the home context and especially for those who are not giving birth for the first time, homebirth is not for everybody. Some women feel more reassured by the hospital context when it comes to an unfamiliar experience, and it should also be noted that nobody can anticipate how they will feel and experience their own bodies in such an extra-ordinary situation. Efforts to make the hospital more home-like and increase privacy are thus a crucial contribution. Yet even more important is the focus on the quality of care because as human beings, we are most affected by our relations to other humans, especially in a situation that is uncanny and in which we are dependent on the help of others. Reflections on discourse, listening, care, and compassion are thus very important (and should ideally play a key role in midwives’ training and professional development).^17^

Being with somebody who is undergoing an experience of uncanniness and passivity requires compassion.^18^ My relationship to the Other does not necessarily require for me to project how I would feel in the situation of giving birth; rather, it can arise from the realisation that this situation is a special challenge. This challenge is always experienced in a unique way and yet such that some structural characteristics such as uncanniness and passivity can be identified. The description of the birth experience provided here has confirmed the crucial role of midwives and other health professionals who help the birth-giving process. Initially, there might be clashes: between the woman facing a radically strange and inconceivable experience, and the midwife to whom such experiences are normal events (and who comes herself as a stranger). Being with the Other in this kind of situation requires being there for them in such a way as to not leap in but leap ahead, liberating the Other to undergo this unimaginable yet possible experience of uncanniness and wonder. Liberating the Other through care means listening to their imagined birth scenario and trying to accommodate them whenever possible, including choice of birth place. But it also involves inspiring their imagination by giving them more options, outlining choices in terms of places and positions, without being offended if these options are not taken up.

It means trying to make the Other comfortable in the midst of uncanniness, passivity, and waiting. This is a tremendously difficult task, and one that involves knowing how to be there for the Other with and without words. It involves patience, silence, listening, flexibility, and other modes of being with the Other by being there for them. But if the midwife establishes successful care relations via discourse in the wider sense developed here, she might actually be experienced as an angel of sorts who comes as a guide or guardian in the encounter with the uncanny and makes the seemingly impossible possible.

## Footnotes

1 Speaking from personal example or auto-ethnographic observation: it was because of this uncertainty around the ‘when’ that I had to overcome my resistance to mobile phones during my first pregnancy. For any subsequent pregnancy, the need to create alternative arrangements for the older sibling(s) enhances the sense of uncertainty.

2 In relation to the discussion of homebirth, this fact will play an important role (see below). There are countries where a midwife can be selected beforehand, such as Germany; yet even so, there are limitations: not only is this usually an expensive option, but there even still remains some unpredictability since the midwife might be with a different woman at the time, or otherwise unavailable, which is again a function of the ‘when’ being unpredictable. By and large, it thus holds true that the midwife is another unknown factor.

3 Yet it can also be fear-alleviating for those who find the presence of medical equipment comforting, and generally, we will come to conclude that the quality of care matters more than the birth-setting, as far as alleviating fears is concerned.

4 In this study, there were 5.3 of such incidences in 1000 births for obstretic units versus 9.3 for home, both for nulliparous women. The adjusted odds ratio thus comes to 1.75.

5 See esp. Thomson et al. (2011).

6 In the UK context, the midwife will only by chance be the same midwife who provided antenatal care, or else, the pregnant woman would need to sign up for a private independent midwifery service rather than NHS midwives. These tend to be costly and are not available in all areas. The expense of having familiar midwives attend homebirths differs from country to country, and can also change radically, as in Germany where a change in insurance provision suddenly doubled the expense and thus made homebirth much less feasible as an option. In the UK, the President of the Royal College of Midwives, Lesley Page, has been lobbying for what she calls “1–1 birthcare” for a long time, and it seems clearly outside of the possibilities of the National Health Service.

7 See Derrida (2000).

8 This dimension is explicitly mentioned in the NHS guidelines for homebirths, and while the recommendation to provide a snack initially seems unproblematic, this can well change under the birth circumstances. (Auto-ethnographic observation: I did not appreciate having my partner prepare tea and snacks in the kitchen while I wanted to have him around for back massage during contractions).

9 Auto-ethnographic observation: I felt obligated to switch the TV off (even though I had identified a good episode of ‘Monk’ with my favourite assistant of his, Sharona) when the midwives arrived, partly because I felt it would otherwise have been hospitable to ask about entertainment preferences, and I was not in a position to discuss my programme choices. Plus, when there is the possibility or actuality of examination, visual entertainment also feels out of place.

10 Derrida explores “unconditional” hospitality, in contrast to “conditional” hospitality. His terms operate somewhat differently, especially since unconditional hospitality designates a kind of general and absolute welcome, irrespective of where the stranger is coming from. For the relationship discussed in the current article, the stranger would always be a midwife and thus not a stranger in the most universal sense. At the same time, there are certain parallels between fundamental hospitality as discussed here and unconditional hospitality in Derrida’s sense; for example, both are in some sense impossible or cannot be fully achieved. See Derrida (2000).

11 See Lundgren (2011).

12 In that sense, our conclusions coincide with Brocklehurst’s recommendation, and it should indeed be noted that it takes financial, political, and communal efforts to provide women with these choices.

13 The statement that the body plays no intrinsic role in waiting does not exclude that the body might be important in certain experiences of waiting (such as standing around and waiting); yet for a general description of waiting as attempted here in its briefest form, the body is not crucial because it does not always play a role in waiting. The question as to whether time-consciousness does not inevitably need to be embodied operates on a different level (namely, (phenomeno-)logical necessity rather than experiential evidence).

14 Auto-ethnographic observation: I decided that the waiting process during early stages of labour would call for a detective novel that draws the reader in. My choice was Bernhard Schlink (*Self’s Punishment*); he served me well.

15 The general implications of Heideggerian care for healthcare relationships are explored in Mallia (2012), Mallia and Ten Have (2003), and Svenaeus (2011).

16 The significance and difficulty of staying a ‘who’ emerges when women report negative experiences in which it felt like the process was happening without them: “Mentally, I was totally finished. I really wasn’t there any more” (reported in Ayers 2007, p. 258).

17 We have developed an i-learn module to that effect for the Royal College of Midwives, entitled *Communicating Well. Philosophy for Midwives*.

18 For an excellent recent example which offers lots of voices and perspectives, see Byrom and Downe (2015).
